# A Quantum Cascade Laser-Based Multi-Gas Sensor for Ambient Air Monitoring

**DOI:** 10.3390/s20071850

**Published:** 2020-03-26

**Authors:** Andreas Genner, Pedro Martín-Mateos, Harald Moser, Bernhard Lendl

**Affiliations:** 1 Institute of Chemical Technologies and Analytics, TU Wien, Getreidemarkt 9/164, 1060 Vienna, Austria; andreas.genner@tuwien.ac.at (A.G.); harald.moser@tuwien.ac.at (H.M.); 2 Electronics Technology Department, Universidad Carlos III de Madrid, C/Butarque 15, 28911 Leganés, Madrid, Spain; pmmateos@ing.uc3m.es

**Keywords:** quantum cascade laser, infrared, ambient air, wavelength modulation spectroscopy, heterodyne phase sensitive dispersion spectroscopy

## Abstract

A quantum cascade laser-based sensor for ambient air monitoring is presented and five gases, affecting the air quality, can be quantified. The light sources are selected to measure CO, NO, NO_2_, N_2_O and SO_2_. The footprint of the measurement setup is designed to fit in two standard 19” rack (48 cm × 65 cm) with 4 height units (18 cm) whereas one is holding the optical components and the other one contains the electronics and data processing unit. The concentrations of the individual analytes are measured using 2f-Wavelength Modulation Spectroscopy (2f-WMS) and a commercially available multipass gas cell defines the optical path. In addition, CO can also be measured with a dispersion-based technique, which allows one to cover a wider concentration range than 2f-WMS. The performance of this prototype has been evaluated in the lab and detection limits in the range of 1ppbv have been achieved. Finally, the applicability of this prototype for ambient air monitoring is shown in a five-week measurement campaign in cooperation with the Municipal Department for Environmental Protection (MA 22) of Vienna, Austria.

## 1. Introduction

Measuring the air quality has become an important task in analytical chemistry over the last decades and it was mainly caused by the combustion of fossil fuels. While CO_2_ is the primary product of the oxidation process, toxic gases, such as CO, NO, NO_2_ and SO_2_, are formed as well. Measures, such as the desulfurizing of diesel, gasoline or the resulting flue gas already improved the air quality in industrial cities significantly during the last decades [1,2]. A recent example of further efforts to reduce air pollution is the limitation of diesel cars in cities with high NO_x_ levels, which are considered to cause respiratory diseases [3]. Nevertheless, the effectiveness of those measures needs to be evaluated and are therefore in the interest of (non-) governmental air-quality measurement networks. The requirements for such a measurement station are typically regulated in local laws and the globally important regulations, such as the Directive 2008/50/EC (EU), the Clean Air Act (USA) and the Law on Prevention and Control of Air Pollution (China) should be noted.

The instrumental equipment can differ between the measurement stations but in general, it can be said that each analyte/measurement parameter requires different equipment. For example, SO_2_ is measured with UV fluorescence, the NO_x_ detection relies on chemiluminescence and the CO/CO_2_ is quantified with a non-dispersive infrared sensor. One way to reduce the investment/operation costs and the required space of the measurement station is to combine and simplify the equipment/components. As the previously listed analytes are gaseous and rather small molecules, an infrared based technique could be applied. In infrared spectroscopy, Fourier transformation-based spectrometers are still the “golden standard” and cover the whole mid-IR. However, the typical concentrations of the target analytes require an optical path length >20 m, which is difficult to achieve in combination with standard Fourier transform infrared (FTIR) equipment.

An alternative is to replace the light source with a collimated semiconductor laser. Beside the higher optical power and the better beam quality, they do not require an interferometer. Instead, the emitted wavelength can be controlled by the temperature of the gain element and the injected current.

Lead–salt diode lasers were the first successful semiconductor based mid-IR diode lasers [4,5,6,7,8,9,10,11], but only a decent number of gas sensors, working in the mid-IR, have been demonstrated and published [12,13,14,15,16,17,18,19]. This has changed with the development of the quantum cascade laser (QCL) [20]. The first devices required liquid nitrogen for cooling and allowed only pulsed operation, but they are currently commercially available in continuous wave mode at room temperature and emit up to several hundreds of mW. So far, numerous measurement techniques with QCLs have been applied to ambient air monitoring—ranging from Tunable Diode Laser Absorption Spectroscopy (TDLAS) [21,22,23], 2f-Wavelength Modulation Spectroscopy (2f-WMS) [24], photoacoustic [25,26,27] photothermal spectroscopy [28], cavity enhanced absorption spectroscopy [29] and also dispersion-based ones [30] have been demonstrated.

In this work, we present a sensor based on 2f-WMS that is capable to quantify the analytes CO, NO, N_2_O, NO_2_ and SO_2_ in the single digit ppbv-range in ambient air. With the exception of the breadboard and a 3d-printed mount for a reference cell, all other parts are off-the-shelf components and easily available. In addition, we show the integration of Heterodyne Phase Sensitive Dispersion Spectroscopy (HPSDS) to cover a significantly higher CO concentration range and present results of a measurement campaign in Vienna.

## 2. Design Considerations for the Multi-Gas Sensor

**Figure 1:**
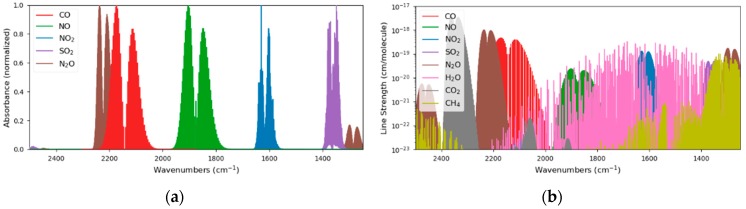
Although the target analytes show nicely separated absorption regions (**a**), the presence of other gases in the ambient air, such as H_2_O, CH_4_ and CO_2_, require a precise wavelength selection of the laser sources (**b**).

Due to the molecular properties of the analytes of interest, their absorption lines are clearly separated, in the range between 4 and 8 µm (Figure 1a) and could easily be quantified with filter-based IR sensors. As the ambient air contains other IR active molecules like H_2_O, CO_2_ or CH_4_ as well, only a few absorption lines are interference-free and suited for quantification with IR spectroscopy. This situation is illustrated in Figure 1b whereas the line strengths of the individual analytes are plotted on a logarithmic scale.

QCLs with an external cavity (EC-QCLs) can already cover a spectral range of >400 cm^−1^ [31], but they are rather unsuited for gas phase measurement. This is caused by the mechanical parts that are required to select the emitted wavelength and mode hops are a common issue. They are mainly used for measuring solids [32,33], liquids [34,35] or polyatomic gasses and vapors with broad absorption bands [36,37]. During the last decades, QCLs with distributed feedback (DFB) gratings [38] have proven as reliable light sources for gas phase spectroscopy. An optical grating, which is etched into the waveguide during the manufacturing process, is responsible for singe mode emission. Subsequently, the emitted wavelength can only be tuned by either changing the injected current or the temperature of the gain element. Common values for this tuning range are a few wavenumbers and usually more than one absorption line of the target analyte is within the accessible range.

As the absorption lines of our target analytes are distributed over the mid IR range and cannot be covered by a single DFB-QCL, multiple lasers are necessary. On the wafer scale level, QCL-arrays in different geometries have been demonstrated [39,40,41,42] and even the integration of infrared detectors is possible [43]. Due to the weak demand of such arrays, they can still be seen as research devices and the preferred way is to employ individually packaged DFB-QCLs that are then combined with dichroic mirrors or beamsplitters.

### 2.1. Spectral Coverage of the QCLs and Absorption Lines of the Analytes

**Figure 2:**
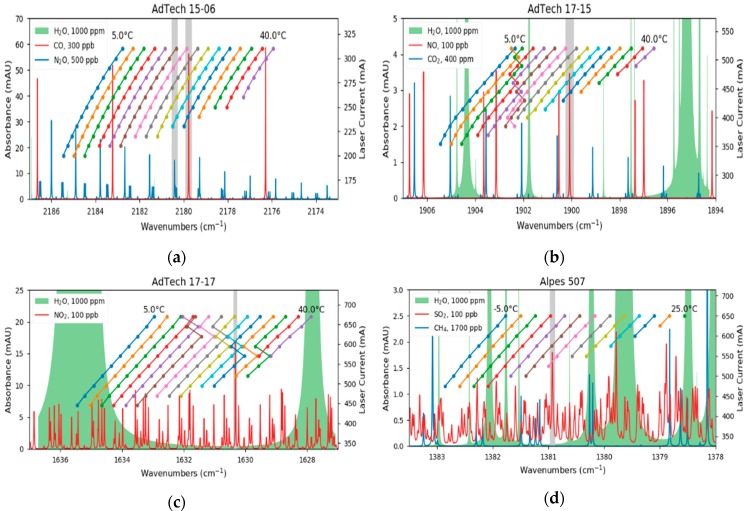
Characterization of the cw-QCLs. While the lasers for CO (**a**) and SO_2_ (**d**) are mode-hop-free, the devices for NO (**b**) and NO_2_ (**c**) have mode-hops within their operation range. In addition, absorption spectra of the analytes and other interfering species are plotted. The grey sections indicate the investigated absorption lines of the analytes.

The DFB-QCLs that are used in this multi-gas sensor are commercially available (AdTech Optics, City of Industry, CA, USA and Alpes Lasers, St. Blaise, CH) and mounted in a high heat load (HHL) package. Each device was characterized with a high resolution FTIR spectrometer (Vertex 80v, Bruker Optics, Ettlingen, DE) whereas spectra were recorded at different gain element temperatures and laser currents. The emitted wavelength, depending on the operation parameters, is plotted in Figure 2a–d and spectra of the target analytes and their major interferences are shown as well. The data is derived from the HITRAN database [44,45] and calculated for a pressure of 100 mbar, room temperature and 76 m optical path length. The laser parameters used for the further experiments are listed in Table 1.

Although the QCLs emit typically single-mode, two devices showed mode hops during the characterization. In the case of the QCL for NO, this occurs at laser parameters where the analyte is not absorbing and therefore not relevant (Figure 2b). In contrast, the laser for NO_2_ has a mode hop at the same wavelength as the probed absorption line (Figure 2c). However, the thermal and electrical parameters of the gain element allowed us to find suitable laser parameters where the mode hop does not influence the measurements.

### 2.2. Opto-Mechanics

**Figure 3:**
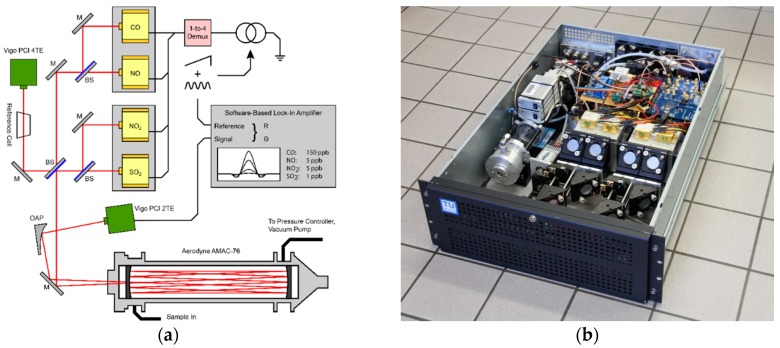
Schematic layout of the individual components for the 2f-WMS based sensor (**a**) and the assembly of the optical setup in a standard 19” server rack (**b**). The laser driver, data-acquisition-card, power supplies and the measurement PC are installed in a second rack (not shown).

The laser beams were collimated and then directed with gold mirrors (PF-10-M03, Thorlabs, Newton, NJ, USA) to the first beam splitter stage (CaF_2_, BSW510, Thorlabs, Newton, NJ, USA). Here, two lasers that were on the same aluminum heat sink were combined. A third beam splitter allowed us to combine the beams from the first stages. Approximately 45% of the light was redirected with a flat mirror (PFE10-M01, Thorlabs, Newton, NJ, USA) into the 76 m long Herriott-type multipass gas cell (AMAC 76, Aerodyne, USA) and then focused (MPD149-M01, Thorlabs, Newton, NJ, USA) onto a thermoelectrically cooled MCT detector (PCI-2-TE-12, 200 MHz bandwidth, Vigo Systems, Ozarow Mazowiecki, PL). The other half can be used to track the laser parameters by passing a gas reference cell (Wavelength References, Corvallis, OR, USA) and an additional reference detector (PCI-4TE-9, 20 MHz bandwidth, Vigo Systems, Ozarow Mazowiecki, PL). The overall dimensions of the optical setup were 45 cm × 65 cm, making it perfectly fit in a 19″ server rack (IPC 4U-4129-N, Inter-Tech Elektronik, Langenhagen, DE), as shown in Figure 3.

### 2.3. 2f-Wavelength Modulation Spectroscopy

The prototype is based on Wavelength Modulation Spectroscopy (WMS), which is closely related to the TDLAS. Here, the laser is tuned over an absorption line (e.g., by changing the injected current, tuning an external cavity grating, etc.) and attenuated by the presence of the analyte. After passing the gas measurement cell, the light hits a detector and its signal can directly be used for the Beer–Lambert relation. In contrast, WMS uses a sine wave on top of the slow sawtooth ramp and a lock-in amplifier processes the received detector signal. An ideal lock-in-amplifier recovers only the signal components that arise from the sine wave modulation and suppresses all other frequencies with a low pass filter. It is therefore perfectly suited to extract the desired signals from noisy environments. By demodulating at the harmonics of the sine wave, one gains information on the shape of the absorption line and the peak height of the second harmonic is proportional to the absorption of the analyte. The noise at the maximum of the 2f-sepctrum is further reduced by fitting the center of the spectrum with a parabolic function.

### 2.4. Electronic Parts

A data acquisition card (NI 6366, National Instruments, Austin, TX, USA) produced the control signal for the laser driver (QCL 1000 OEM, Wavelength Electronics, Bozeman, MT, USA). It consisted of a 1 Hz sawtooth ramp with a 20 kHz sine wave on top. The rather slow sawtooth function was responsible for tuning the emitted wavelength over the absorption line and the sine wave was responsible for the wavelength modulation. Each QCL requires specific amplitudes of the individual signal components as the material properties and dimensions of the gain chips differ. Considering the additional costs and the required space of individual laser drivers, a low-cost 1-to-4 demultiplexer (4 channel relay module, SainSmart Technology, Lenexa, KS, USA) redirects the laser current to the desired QCL. Each laser was protected with a dedicated fuse and an electrostatic discharge (ESD) absorber (LA44-2000, Lasorb, Sanford, FL, USA).

The detector signal was digitized with the data acquisition (DAQ) card at 1 MSPS and demodulated with a software-based lock-in-amplifier (120 dB/decade, time constant=1 ms, FIR). The demodulation was set to the second harmonic of the sine wave modulation (1f = 20 kHz) and, as the laser was tuning over an absorption line with 1 Hz, a 2f-WMS spectrum was recorded every second. The hardware for the signal processing, the measurement PC and the required power supplies were installed in a second, dedicated 19” rack (IPC 4U-4129-N, Inter-Tech Elektronik, Langenhagen, DE) and the overall power consumption was 280 W.

Beside the high precision laser driver, DAQ card and demultiplexer, the sensor requires an accurate temperature control of the laser gain chips. Therefore, each HHL package had its own thermoelectric cooling (TEC) controller (TEC 1091, Meerstetter Engineering, Rubigen, CH), which red out an NTC and adjusts the current for the Peltier element. An additional dual-channel TEC controller (TEC 1122, Meerstetter Engineering, Rubigen, CH) was installed to stabilize the temperature of the aluminum plate where the HHL packages were mounted. The pressure in the multipass gas cell was adjusted with a pressure controller (GSP-C5SA, Vögtlin Instruments, Aesch, CH) and set to 100 mbar. To avoid mechanical vibrations, the required vacuum pump (N860.3FT.40.18, KNF Neuberger, Freiburg, DE) was placed next to the prototype.

## 3. Experiments with 2f-Wavelength Modulation Spectroscopy

### 3.1. Lab Evaluation

**Figure 4:**
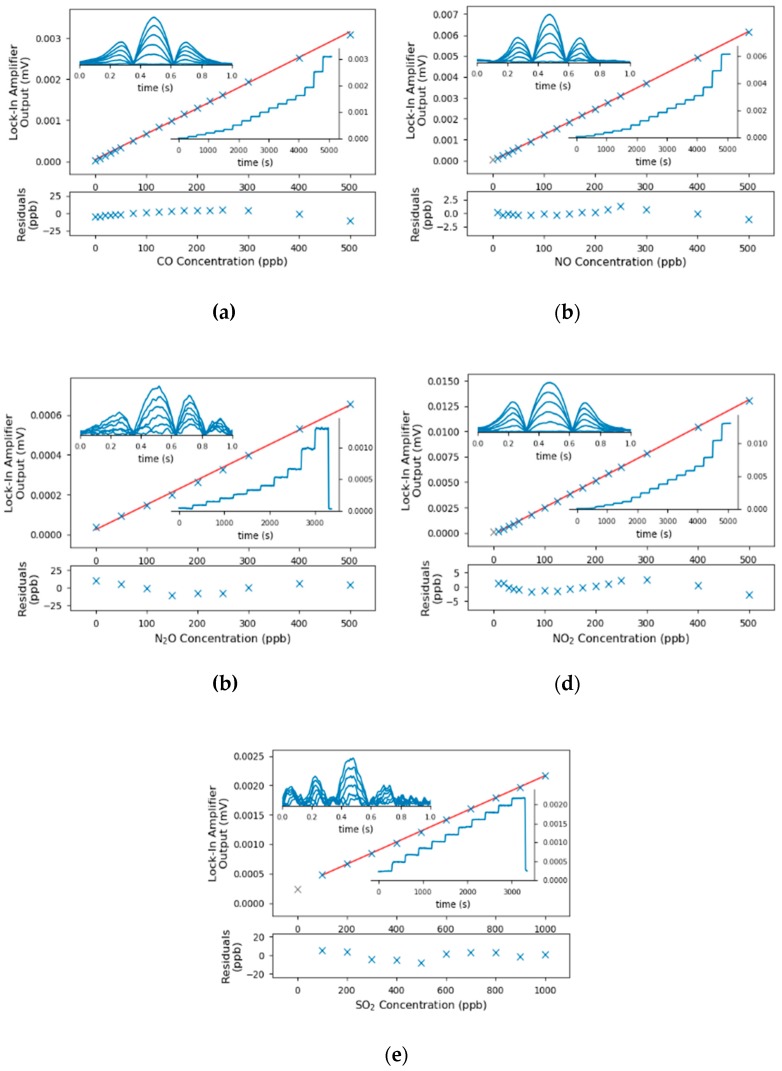
Calibration curves (center), the associated residuals (bottom), the peak signal of the lock-in-amplifier and selected spectra (insets) for the accessible pollutants. The calibration points indicated in grey have been identified as outliers and were excluded from the linear calibrations. The concentrations for the presented spectra in (**a**–**d**) were 0, 100, 200, 300, 400 and 500 ppbv; the concentrations for SO_2_ (**e**) were 0, 200, 400, 600, 800 and 1000 ppbv.

The sensor was calibrated with an inhouse built gas mixing rig whereas two mass flow controllers (GSC-B9TS-BB23, Vögtlin Instruments, Aesch, CH) defined the mixing ratio of the gas from the individual test gas bottle and nitrogen (N_2_ 5.0, Messer Austria, Gumpoldskirchen, AT). The concentration was increased in a stepwise manner, covering the range between 0 and 500 ppbv. The flow rate was set to 1 L/min and each concentration step was held for 5 min to exchange and stabilize the sample composition in the gas cell. As the gas sensor allows only sequential quantification of the analytes, the QCLs were not switched during the calibration runs. The fitted peak amplitude of the 2f-WMS-spectrum were averaged over 10 s and the resulting graphs are shown in Figure 4. The minimum detection limits were calculated from the signal to noise ratios at 100 ppbv and are listed in Table 2.

One can see from the calibrations, that the LODs for SO_2_ and N_2_O were significantly higher than the other components. In the case for N_2_O, the increased noise can be traced back to fringes as the mirrors and detector signal were optimized for the laser parameters to quantify CO. For the SO_2_, in contrast, the interference free absorption lines of SO_2_ were in general rather weak, which resulted in higher detection limits.

**Figure 5:**
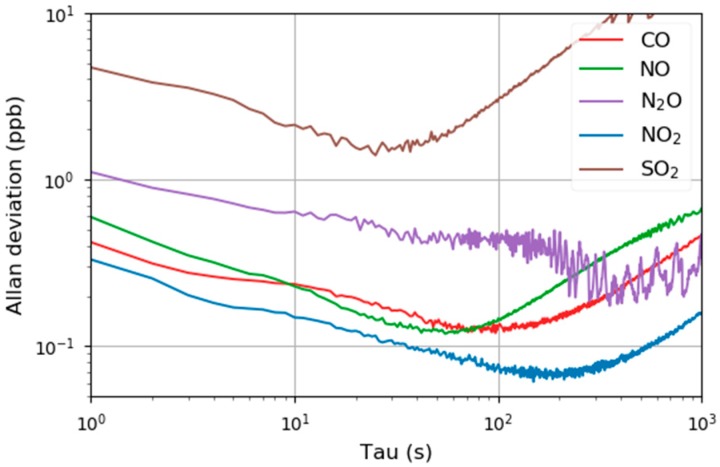
Allan–Werle plot for five pollution relevant analytes that can be detected with the multi-gas sensor.

The temporal stability of the sensor was evaluated by measuring a constant concentration of the analyte for 1 h (CO, NO, N_2_O and NO_2_: 100 ppbv, SO_2_: 500 ppbv). The Allan–Werle variance (Figure 5) retrieved an optimum integration time between 30 and 100 s. This result is in the same region as other publications [47,48,49] and it is assumed that thermal drifts in the lab (air conditioner) limit the maximum integration time.

### 3.2. Field Evaluation of CO, NO and NO_2_

**Figure 6:**
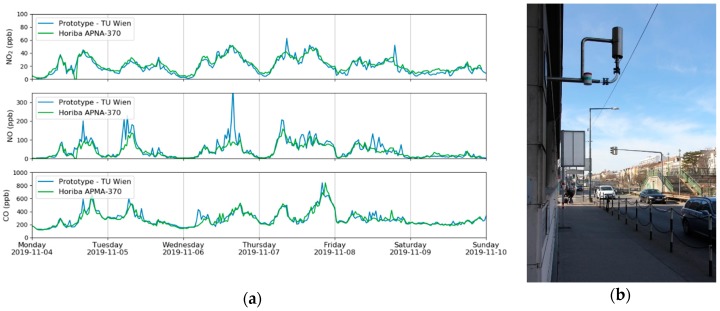
Time series of CO, NO and NO_2_ during one week of the measurement campaign in Vienna. Beside data from the QCL sensor, values from the MA 22 are plotted too (**a**). The intake of the measurement station (**b**) is located close to an intersection of a busy road.

A measurement campaign was carried out in Vienna, Austria, in October/November 2019 and the focus was on the analytes CO, NO and NO_2_. As the sensor can only quantify one component at a time, it was set to monitor one analyte for 10 min and then to switch to the next one. This guaranteed a reasonable measurement duration for each component but also an acceptable interval. Additional reference data was gained at the installation site using commercially available equipment, based on chemiluminescence (NO, NO_2_ and NO_x_, Horiba APNA-370, Kyoto, JP) and non-dispersive IR measurements (CO, Horiba APMA-370, Kyoto, JP). The data from one week is plotted in Figure 6 and one can clearly see that the results of the QCL based sensor were very similar to the values from the reference methods.

While the analytes NO and NO_2_ were always within the linear calibration range of the sensor, several short time events with significantly higher CO concentrations occurred during the field test. They exceeded the linear range of the calibration and the resulting data is therefore not reliable. One solution would have been to modify the hardware by, for example, reducing the optical path length or diluting the sample stream. However, these options have not been applicable in the experimental setup and it was solved by integrating the Heterodyne Phase Sensitive Dispersion Spectroscopy as a second measurement modality.

## 4. Heterodyne Phase Sensitive Dispersion Spectroscopy to Quantify High CO Concentrations

### 4.1. Required Modifications

As one can see from the recorded calibrations, 2f-WMS was perfectly suited for the quantification of low-ppbv-concentrations, but it shows a non-linear behavior at rather high concentrations. In particular, the gained 2f-WMS signal from CO concentrations higher than 1 ppmv was not linear anymore and the calibration would require a non-linear fitting-function. This limitation origins from the Beer–Lambert–Bouguer law and can be solved by changing the measurement technique. Photo-thermal and photoacoustic methods, for example, enable a linear range over several magnitudes, but they require special designed gas cells and cannot be implemented in the existing system. A method that can be easily integrated in this direct absorption-based sensor is Heterodyne Phase Sensitive Dispersion Spectroscopy [50]. Instead of quantifying the attenuation of the laser beam caused by the presence of the analyte, the phase shift of the laser beam, introduced by the dispersion of the gas, is measured.

The basic principle is to superimpose the laser current with a sine wave in the high MHz range, causing the emission of two additional sidebands. They are separated from the emitted center wavelength by the applied modulation frequency. Each optical tone undergoes different refractive indices while passing the gas measurement cell. These optical phase shifts are proportional to the wavelength dependent dispersion of the analyte, its concentration and the optical path length. To detect these phase shifts, the bandwidth of the detector must be in the same range as the modulation frequency. The relevant information is the phase and it is, for a wide concentration range, independent of the amplitude.

**Figure 7:**
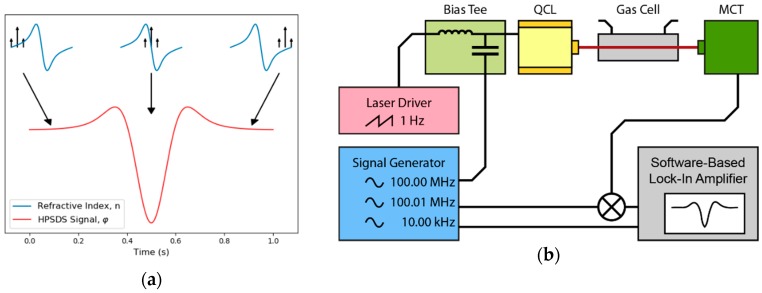
(**a**) The laser source is intensity-modulated and generates an optical three-tone-signal. While the laser is tuned across the absorption line, the phases of the three wavelengths are differently shifted because of the wavelength dependent refractive indices. (**b**) The detector signal is downmixed to a lower frequency and a software-based lock-in-amplifier recovers the phase information.

The required hardware modification involves standard components for radio-frequency experiments that are commercially available. A four-channel frequency source (AD9959, Analog Devices, Norwood, MA, USA) generated a sine wave with 100.00 MHz, which was added to the laser current with a bias-tee (ZFBT-6g+, Mini-Circuits, Brooklyn, NY, USA). As the software-based lock-in-amplifier was not capable to demodulate at the resulting frequency, the detector signal must be downmixed with an analog mixer (ZAD-1-1+, Mini-Circuits, Brooklyn, NY, USA). The required reference signal was slightly higher (100.01 MHz) and synthesized on the second channel of the AD9959. The third channel generated the difference frequency (10 kHz), which allowed us to demodulate the downmixed signal with the software-based lock-in-amplifier (Figure 7). Like TDLAS and 2f-WMS, the absorption line was probed by tuning the center-wavelength of the QCL (1 Hz sawtooth current ramp).

### 4.2. Calibration of CO

**Figure 8:**
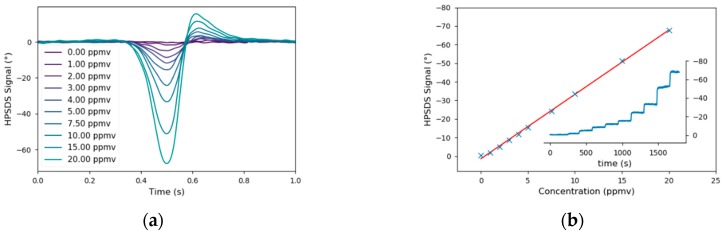
Heterodyne Phase Sensitive Dispersion Spectroscopy (HPSDS) spectra (**a**) from the calibration of CO (**b**). The asymmetry of the spectra is the result of the rather high laser current, which is necessary to achieve a sufficient signal on the detector.

Again, the applicability of this hardware upgrade of the prototype was verified in the lab by recording a calibration of CO in N_2_ whereas a concentration range of 0–20 ppmv was covered. In contrast to the 2f-WMS measurements, the pressure in the gas cell was reduced to 30 mbar. This was necessary because the maximum phase signal is achieved if the modulation frequency is 0.6 times the full width at half maximum (FWHM) of the spectral line [51]. As the electrical bandwidth of the detector and the signal generator are limited, one can only improve the signal by reducing the FWHM of the spectral feature. To compensate the additional phase caused by the cables and optical elements, a HPSDS background spectrum (only N_2_ in the gas cell) was subtracted. Then, the phase at the minimum was used to calculate the linear regression. The HPSDS spectra and calibration curve (R^2^ = 0.9988) were plotted in Figure 8 and the detection limit was 0.08 ppmv.

### 4.3. Field Test and Discussion

**Figure 9:**
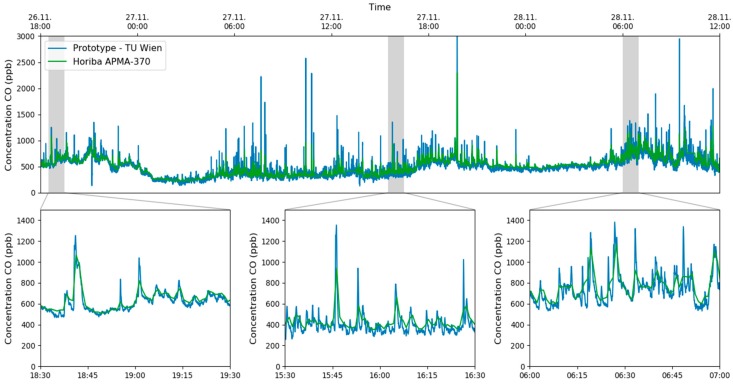
Time series of the CO concentration measured with HPSDS and the data from the NDIR based reference equipment. The responsiveness of setup can be seen in the zoomed in plots.

The applicability of HPSDS for measuring CO in the ambient air has also been tested during the campaign and compared with values from the commercial reference sensor. To show the performance of this advanced technique, only CO has been measured during this 42 h experiment. The retrieved concentrations were compared with the one-minute-average values from the reference equipment and are shown in Figure 9.

Considering the different temporal resolutions of the multi-gas sensor and the reference equipment, one can see that the graphs matched perfectly. The sudden increase of CO at the sampling point of the measurement station was caused by the traffic light-controlled intersection, shown in Figure 6b. One can clearly identify phases with a lower CO concentration during the night (e.g., 27 November 0:00–4:00, 28 November 0:00–5:00) and increased levels during the rush hours in the morning and evening.

## 5. Conclusions

In this paper, we demonstrated a QCL based sensor for monitoring gaseous analytes that strongly contributed to the overall air quality. The electrical and optical components of the setup are commercially available and can be, due to the installation in a standard 19” server rack, easily installed next to commercial ambient air monitors and maybe even replace them in the future. By default, the analytes were measured with 2f-WMS and the characterization in the lab revealed LODs <1 ppbv for the main analytes (CO, NO and NO_2_) and slightly higher values for N_2_O and SO_2_. According to the Allan variance plots, this could be improved by averaging over 30–60 s, however, an integration time of 10 s was used to guarantee a reasonable response time. Comparing the results to other multi-components QCL sensors, it was suspected that the wedged beamsplitters and the lack of not perfectly optimized electronics are the main reasons for the slightly higher detection limits.

In addition, HPSDS was integrated in the sensor that was originally designed for 2f-WMS measurements. The calibration of CO in the lab showed that the linear range of the prototype could be extended to cover concentrations of up to 20 ppmv without replacing the gas measurement cell or installing an additional gas dilution system.

Both techniques, the 2f-WMS and HPSDS, were applied in a measurement campaign in Vienna and the results were in good agreement with the reference values. Subsequently, this QCL-based multi-gas sensor can be seen as a step forward to a new generation of high precision and still very flexible gas monitoring systems.

## Figures and Tables

**Table 1:** Laser parameters used for 2f-Wavelength Modulation Spectroscopy (2f-WMS). The amplitudes of the sine wave modulation were adjusted to achieve the maximum lock-in amplifier signal, according to [46].

Manufacturer	Analyte	Wavenumbers (cm^−1^)	Temperature (°C)	Current Ramp (mA)	Modulation (mA)
AdTech Optics	CO	2179.77	20.85	297.0–300.2	1.0
	N_2_O	2180.42	17.61	297.0–300.2	1.1
AdTech Optics	NO	1900.08	21.09	514.0–520.0	1.6
AdTech Optics	NO_2_	1630.33	27.57	640.2–646.5	2.8
Alpes Lasers	SO_2_	1380.93	3.95	625.0–640.0	5.2

**Table 2:** Linear response and detection limits derived from the calibration for the individual target analytes.

Analyte	Calibration R^2^	SNR at 100 ppbv, 1 Hz Sample Rate	1σ Detection Limit (ppbv), 1 Hz Sample Rate
CO	0.99918	308	0.32
NO	0.99998	224	0.45
N_2_O	0.99798	40	2.51
NO_2_	0.99990	233	0.43
SO_2_	0.99978	71	1.40
